# Enriching Glucoraphanin in *Brassica rapa* Through Replacement of *BrAOP2.2/BrAOP2.3* with Non-functional Genes

**DOI:** 10.3389/fpls.2017.01329

**Published:** 2017-08-02

**Authors:** Zhiyuan Liu, Jianli Liang, Shuning Zheng, Jifang Zhang, Jian Wu, Feng Cheng, Wencai Yang, Xiaowu Wang

**Affiliations:** ^1^Institute of Vegetables and Flowers, Chinese Academy of Agricultural Sciences Beijing, China; ^2^College of Horticulture, China Agricultural University Beijing, China

**Keywords:** *Brassica rapa*, glucosinolate, glucoraphanin, *BrAOP2*, Marker-assisted backcrossing

## Abstract

Sulforaphane, the hydrolytic product of glucoraphanin glucosinolate, is a potent anticarcinogen that reduces the risk of several human cancers. However, in most *B. rapa* vegetables, glucoraphanin is undetectable or only present in trace amounts, since the glucoraphanin that is present is converted to gluconapin by three functional *BrAOP2* genes. In this study, to enrich beneficial glucoraphanin content in *B. rapa*, the functional *BrAOP2* alleles were replaced by non-functional counterparts through marker-assisted backcrossing (MAB). We identified non-functional mutations of two *BrAOP2* genes from *B. rapa*. The backcross progenies with introgression of both non-functional *braop2.2* and *braop2.3* alleles significantly increased the glucoraphanin content by 18 times relative to the recurrent parent. In contrast, replacement or introgression of single non-functional *braop2.2* or *braop2.3* locus did not change glucoraphanin content. Our results suggest that replacement of these two functional *BrAOP2* genes with non-functional alleles has the potential for producing improved *Brassica* crops with enriched beneficial glucoraphanin content.

## Introduction

Glucosinolates are a group of specialized secondary metabolites which are rich in nitrogen and sulfur in the order Brassicales (Halkier and Gershenzon, 2006; Sønderby et al., 2010). Glucosinolates and their hydrolytic products are well-known for their bioactivities such as, fungicidal, bactericidal, and cancer-preventive attributes (Fahey et al., 2002; Bones and Rossiter, 2006; Zhang et al., 2006; Clay et al., 2009; Gimsing and Kirkegaard, 2009). For human and animal consumption, some glucosinolates are beneficial while others are detrimental. For example, sulforaphane, the hydrolysis product of glucoraphanin, has been reported to possess cancer-preventive attributes because it can modulate phase I and II detoxification enzymes, thereby affecting cancer development (Mithen, 2001; Mithen et al., 2003; Talalay et al., 2007). In contrast, progoitrin is mainly enriched in rapeseed and is anti-nutritional because its breakdown product has toxic effect on animal and human consumption (Griffiths et al., 1998). Since *Brassica* species encompass many important vegetable, oilseed, and fodder crops, it is desirable to increase beneficial glucosinolate (glucoraphanin) and reduce the detrimental glucosinolate (progitrin) in *Brassica* crops to improve their economic and nutritional values and further enhance the potency of their anticancer attributes.

Biosynthesis of glucosinolates is rather complex process in Arabidopsis and more than 50 genes are involved in, including three major steps: side chain elongation core structure modification, and side chain modification (Kliebenstein et al., 2005; Halkier and Gershenzon, 2006; Sønderby et al., 2010; Li et al., 2011; Redovniković et al., 2012). Compared with Arabidopsis, glucosinolate biosynthesis in *Brassica* species is more complex and 102 orthologous glucosinolate-related genes and 105 glucosinolate-related genes were identified in *B. rapa* and *B. oleracea* using comparative genomic analysis, respectively Wang H. et al., 2011; Liu et al., 2014). This is because the *Brassica* genome experienced the whole genome triplication and chromosomal rearrangements since its divergence from Arabidopsis.

The structural diversity of glucosinlates is due to variation in side chain length and secondary modification (Kliebenstein et al., 2001a; Halkier and Gershenzon, 2006). The *AOP* family of genes including *AOP1, AOP2*, and *AOP3* are mainly responsible for secondary modification of the side chain (Hall et al., 2001; Kliebenstein et al., 2001b; Neal et al., 2010). *AOP1* gene is reported as the ancestral gene of *AOP2* and *AOP3*, and its function remains unknown (Wittstock and Halkier, 2002; Neal et al., 2010). *AOP2* is responsible for the conversion of methylsulfinylalkyl glucosinolates to alkenyl glucosinolates (Kliebenstein et al., 2001b). The step is involved in 4C aliphatic glucosinolate, referring glucoraphanin to gluconapin. *AOP3* also show a weak conversion of methylsulfinylalkyl glucosinolate to hydroxyalkyl glucosinolate (Kliebenstein et al., 2001b). Moreover, pervious reports have shown that the accumulation of methylsulfinylalkyl glucosinolates correlate with the expression of a functional *AOP2* and *AOP3* gene in Arabidopsis (Kliebenstein et al., 2001b). These studies indicate that the expressional and functional changes of *AOP2* and *AOP3* genes could affect the accumulation of beneficial glucosinolate (glucoraphanin).

The species *B. rapa* encompass a great array of vegetables, such as, Chinese cabbage, pakchoi, mizuna, narinosa, turnip, and many other leafy vegetables (Zhao et al., 2005). They play an important role in daily diet in many regions of the world, particularly in Asia. However, there are only trace amounts or undetectable levels of beneficial glucoraphanin in *B. rapa* (Padilla et al., 2007; Lou et al., 2008; Kim et al., 2010). In contrast, high concentrations of glucoraphanin have been detected in *B. oleracea* vegetables such as, broccoli, kale, Chinese kale, Brussels sprouts, and purple cauliflower (Fahey et al., 1997; Liu et al., 2012). Further studies have revealed that both *B. rapa* and *B. oleracea* contain three *AOP2* homolog genes but no *AOP3* homolog genes because *Brassica* genome experienced the whole genome triplication (Li and Quiros, 2003; Wang H. et al., 2011; Liu et al., 2014). In *B. oleracea*, two *BoAOP2* genes (*BoAOP2.2* and *BoAOP2.3*) are non-functional as a result of premature stop mutations, while three *BrAOP2* copies are functional in *B. rapa* Wang H. et al., 2011; Liu et al., 2014; Zhang et al., 2015). This is the generally accepted reason for the abundance of glucoraphanin in *B. oleracea*, but not in *B. rapa*. In addition, silencing *AOP* genes (*GSL-ALK*) through RNA interference (RNAi) of *B. napus* and *B. juncea* resulted in accumulation of the content of glucoraphanin, and reduction of detrimental glucosinolate (progitrin) in seeds, respectively (Liu et al., 2012; Augustine and Bisht, 2015). Accordingly, it is possible to increase the beneficial glucoraphanin concentration through genetic manipulation of three *BrAOP2* genes in *B. rapa* crops. However, RNAi-mediated gene knocking down or CRISPR/Cas9-mediated gene knocking out remains a challenge since the inefficient Agrobacteria-mediated transformation in *B. rapa*. Moreover, no mutations of loss-of-function *AOP2s* have yet been found in *B. rapa* or in any *Brassica* species other than *B. oleracea*. In this study, we identified natural non-functional mutantions of two *BrAOP2* genes from “R-O-18” and then performed marker assisted backcross breeding to substitute functional *BrAOP2* gene locus in “L58”with non-functional alleles to increase the beneficial glucosinolate (glucoraphanin) in *B. rapa*. The advanced backcross progenies carrying non-functional *BrAOP2* gene loci were screened with gene specific markers and analyzed for glucosinolate profile and content. Our findings have potential applications in producing glucoraphanin-enriched *B. rapa* crops.

## Materials and methods

### Plant materials for glucosinolate analysis

In the current study, we grew 70 *B. rapa* accessions (Table S1) with various morphological types during autumn 2014. These accessions included the following morphotypes: ssp. *pekinensis* (Chinese cabbage), ssp. *chinensis* (Pakchoi), ssp. *rapifera* (Turnip), ssp. *trilocularis* (Yellow sarson), ssp. *Narinosa* (Wutacai), ssp. *chinensis* var. *tai-tsai* Lin (Taicai), ssp. *parachinensis* (Caixin), ssp. *chinensis* var. *purpurea* Bailey (Zicaitai), ssp. *Perviridis* (Komatsuna), ssp. *nipposinica* (Mizuna), ssp. *broccoletto* (Broccoletto), and ssp. *Oleifera* (Oil).

For all experiments, seeds were first planted in seedling trays. After 20 days, the seedlings were transplanted to larger trays in a greenhouse or field. The youngest leaves of each plant were collected at the rosette stage, immediately frozen in liquid nitrogen and stored in −80°C freezers until freeze-dried with a vacuum freeze dryer. 200 mg of freeze-dried sample of each accession was used for glucosinolate extraction and measurement by high performance liquid chromatography (HPLC). Three biological replicates for each accession were analyzed in independent experiments.

### Plant materials for backcross breeding scheme

The *B. rapa* “R-O-18” (ssp. *trilocularis*) containing high content of beneficial glucosinolate (glucoraphanin) was used as the donor parent, and “L58” (ssp. *parachinensis*) with a lower content of beneficial glucosinolate (glucoraphanin) as the recurrent parent. The two parents were hybridized to produce F_1_, and then backcrossed with “L58” to produce a large BC_1_ population. In the BC_1_population, individual plants with heterozygous locus of *BrAOP2.2* and *BrAOP2.3* was identified for further screening (foreground selection). Then, the individual line with the highest recovery rates was selected with the markers distributed in 10 chromosomes (background selection). When we selected the indidual plant BC_1_-018 with the highest recovery rates from BC_1_ generation, the tissue culture techniques was used for rapid propagation to make sure we could obtain adequate next generation seeds.

In the BC_2_ generation, the same method was employed to select the individual lines with the highest recovery rates. Selected BC_2_ plants were self-pollinated for selection of homozygous loci. These selective BC_2_S_1_were again self-pollinated to produce BC_2_S_2_ for further analysis (Figure S1). All crosses were performed in a greenhouse at the Chinese Academy of Agricultural Sciences (Beijing, China).

### Development of functional marker of *BrAOP2.2* and *BrAOP2.3*

The SNP C/T (+499) of *BrAOP2.2* was converted to KASP assays following instruction from the manufacturer's instructions (http://www.lgcgroup.com/LGCGroup/media/PDFs/Products/Genotyping/KASP-genotyping-chemistry-User-guide.pdf). Primer sequences are listed in Table S2.

In this study, we used 5 μl KASP assay polymerase chain reaction (PCR) mix, which includes 2.5 μl of 2 × KASP Master mix (LGC Genomics, Beverly, MA, USA) of primer assay mix and 2.5 μl genomic DNA at a concentration of 15 ng/μl. The initial PCR mix was performed in a Gene Amp PCR System 9700 (Applied Biosystems, Foster City, CA, USA). The post-PCR fluorescent endpoint readings were carried out using an ABI 7900HT RealTime PCR System (Applied Biosystems, Foster City, CA, USA). The PCR conditions were determined according to the manufacturer's instructions (http://www.lgcgroup.com/LGCGroup/media/PDFs/Products/Genotyping/KASP-genotyping-chemistry-User-guide.pdf).

The insert sequence (+788) of *BrAOP2.3* was verified through InDel assays. The PCR was conducted using the specific primer (Table S2) and the PCR products were resolved on 1.5% agarose gels.

The KASP assays and InDel assays were used to screen and select in the marker-assisted selection.

### Molecular marker analysis

Marker-assisted foreground selection of *BrAOP2.2* and *BrAOP2.3* in backcross progenies were performed using KASP assays and InDel assays, respectively. For marker-assisted background selection, InDel markers were used to calculate the recovery rates of segregants and to select the individual plants with the closest genetic background to the recurrent parent. The InDel primers come from a published linkage map based on an “L58” × “R-O-18” RIL population (Sun et al., 2016). Using the combination of a genetic and a physical map, a total of 100 InDel markers evenly distributed from 10 chromosomes were selected in the BC_1_ generation and 33 InDel markers were used in the BC_2_ generation (Figure S2; Table S3).

### Leaf glucosinolate measurement by HPLC analysis

Glucosinolate profiles of 70 *B. rapa* accessions were determined by HPLC as described by He et al. (2002) with minor modifications. The desulfo glucosinolates were verified based on a comparison of UV absorption spectra and retention times (Brown et al., 2003). The concentration of glucosinolate was calculated with an internal standard.

### Enzyme assays of BrAOP2 protein

The full-length cDNA of *BrAOP2.1, BrAOP2.2*, and *BrAOP2.3* in “R-O-18” were ligated into the pET32a (Novagen, Madison, WI, USA) vector system. The recombinant plasmids were expressed in *E. coli* Transetta (TransGen Biotech, Beijing, China) induced by 0.5 mM IPTG at 16°C for 12–14 h. The crude protein was purified with BugBuster®Ni-NTA His Bind Purification Kit following the manufacturer's instructions (Novagen, EMD Chemicals, CA, USA). The purified protein was confirmed using SDS-PAGE analysis, and then was used for enzyme activity assays as described by Zhang et al. (2015).

### Statistical analysis

Multiple comparisons were used to analyze the variation of glucoraphanin content from advanced backcross progenies (**Figure 4**) using one-way ANOVA with Tukey *post-hoc* test (*P* < 0.05) by SPSS v.19.0 software.

## Results

### Glucosinolate analysis in *B. rapa*

We analyzed glucosinolate from a collection of 70 *B. rapa* accessions, representing 10 morphotypes including Chinese cabbage, Caixin, Komatsuna, Pakchoi, Mizuna, Taicai, Turnip, Wutacai, Yellow sarson, and Zicaitai using HPLC (Table S1). A total of eight types of glucosinolates were detected including four aliphatic glucosinolates (progoitrin, gluconapin, glucobrassicanapin, glucoraphanin) and four indolic glucosinolates (glucobrassicin, 4–methoxyglucobrassicin, neoglucobrassicin, 4–hydroxyglucobrassicin; Table 1). Of these, aliphatic glucosinolate gluconapin and glucobrassicanapin were detected in almost all of the accessions and represented the major glucosinolates, which is consistent with previous studies (Padilla et al., 2007; Kim et al., 2010). The highest total glucosinolate contents (16.22–48.87 μmol g^−1^ DW) and major aliphatic glucosinolates (gluconapin and glucobrassicanapin) were observed in Turnip. A slightly higher content of indolic glucosinolates was found in Chinese cabbage compared with other types. Importantly, out of eight glucosinolates, seven were identified in all accessions except the beneficial glucosinolate (glucoraphanin) was only found in Yellow sarson.

**Table 1:** Glucosinolates in leaves of *B. rapa* (μmol g^−1^ DW).

**Type**	**Aliphatic GSLs**				**Total aliphatic GSLs**	**Indolic GSLs**				**Total indolic GSLs**
	**PRO**	**NAP**	**GBN**	**GRA**		**GBC**	**4ME**	**NEO**	**4OH**	
Chinese cabbage	1.21 ± 0.77	0.91 ± 1.51	1.16 ± 1.45	nd	3.28 ± 2.91	0.98 ± 0.61	0.37 ± 0.19	0.30 ± 0.28	0.11 ± 0.10	1.76 ± 0.71
Caixin	1.02 ± 1.53	5.64 ± 5.20	0.74 ± 0.40	nd	7.39 ± 6.84	0.25 ± 0.13	0.12 ± 0.05	0.18 ± 0.16	0.05 ± 0.03	0.61 ± 0.32
Komatsuna	0.60 ± 0.07	10.20 ± 0.20	4.53 ± 5.28	nd	15.33 ± 5.55	0.39 ± 0.26	0.11 ± 0.01	0.31 ± 0.17	0.07 ± 0.04	0.88 ± 0.47
Pakchoi	0.85 ± 0.20	2.63 ± 2.33	0.87 ± 1.14	nd	4.35 ± 2.97	0.28 ± 0.12	0.14 ± 0.08	0.20 ± 0.11	0.08 ± 0.08	0.70 ± 0.20
Mizuna	0.37 ± 0.04	18.50 ± 8.00	1.19 ± 0.11	nd	20.06 ± 8.27	0.39 ± 0.12	0.17 ± 0.09	0.29 ± 0.18	0.08 ± 0.03	0.92 ± 0.42
Taicai	1.63 ± 0.79	3.11 ± 1.29	3.64 ± 1.43	nd	8.38 ± 3.05	0.48 ± 0.27	0.10 ± 0.03	0.56 ± 0.32	0.19 ± 0.09	1.32 ± 0.50
Turnip	0.44 ± 0.25	24.58 ± 10.70	7.49 ± 3.73	nd	32.51 ± 13.38	0.46 ± 0.06	0.15 ± 0.12	0.30 ± 0.07	0.13 ± 0.03	1.04 ± 0.10
Wutacai	0.71 ± 0.07	2.35 ± 2.78	2.02 ± 2.52	nd	5.09 ± 5.37	0.71 ± 0.01	0.10 ± 0.01	1.01 ± 1.07	0.07 ± 0.02	1.90 ± 1.06
Yellow sarson	0.60 ± 0.09	22.96 ± 0.35	0.22 ± 0.15	2.87 ± 1.82	26.66 ± 2.04	0.09 ± 0.03	0.07 ± 0.01	0.14 ± 0.06	0.02 ± 0.01	0.32 ± 0.06
Zicaitai	0.46 ± 0.53	2.00 ± 2.00	2.04 ± 1.33	nd	4.50 ± 2.59	0.21 ± 0.19	0.12 ± 0.10	0.38 ± 0.54	0.12 ± 0.07	0.83 ± 0.74

*PRO, progoitrin; NAP, gluconapin; GBN, glucobrassicanapin; GRA, glucoraphanin; GBC, glucobrassicin; 4ME, 4–methoxyglucobrassicin; NEO, neoglucobrassicin; 4OH, 4–hydroxyglucobrassicin. GSLs, glucosinolates. nd, not detected. Each value is the mean ± standard deviation*.

### Sequence analysis of three *BrAOP2* genes in Yellow sarson

**Figure 1:**
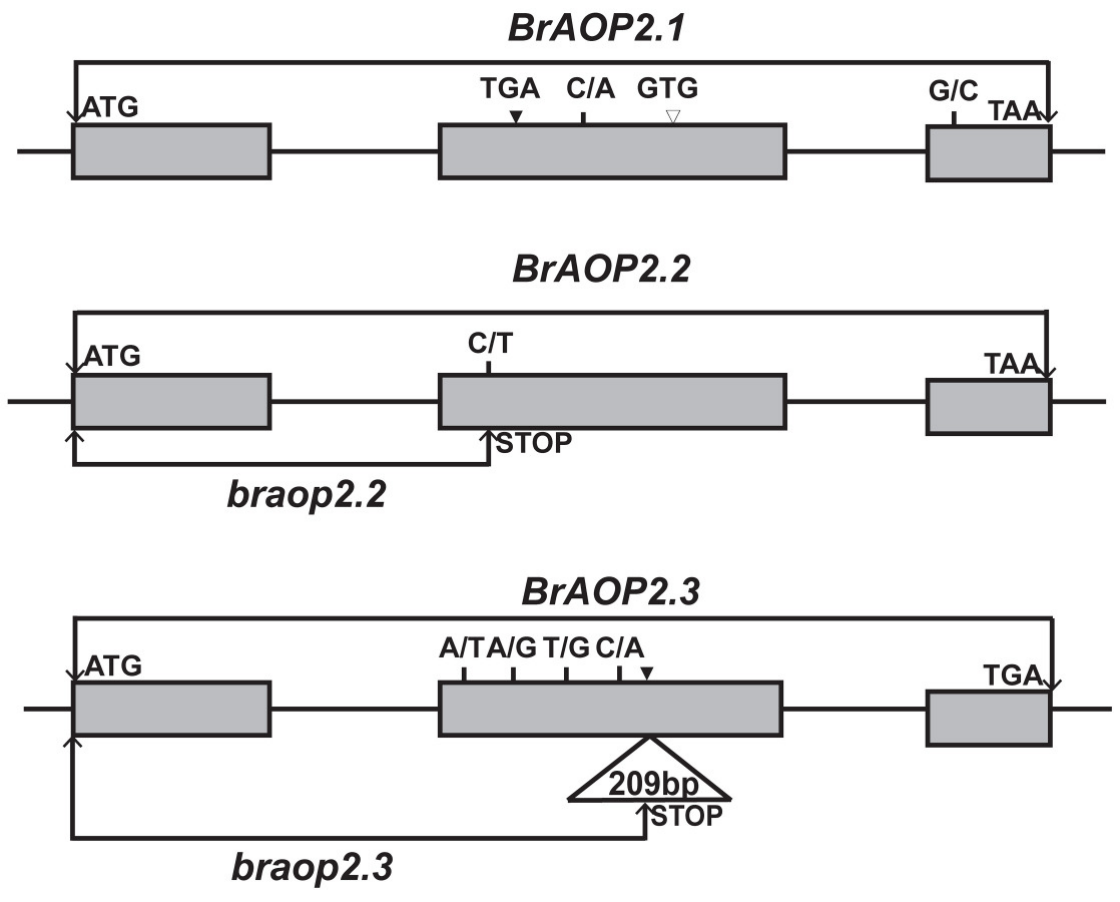
Schematic representation of the sequence variations of *BrAOP2.1, BrAOP2.2*, and *BrAOP2.3* genes from “R-O-18” compared with “Chiifu-401/42.” Gray boxes represent exons, while lines denote introns. Short vertical lines show the non-synonymous SNP variations, and solid triangles and empty triangles indicate insertion and deletion variations, respectively. The SNP C/T at exon 2 (position + 499) of *BrAOP2.2* causes a premature termination codon mutation named *braop2.2*, and a 209-bp insertion in exon 2 (position + 788) of *BrAOP2.3* results in a frame shift and generates a premature stop product named *braop2.3*.

To investigate whether the high accumulation of glucoraphanin in Yellow sarson is due to the non-functional mutation of *BrAOP2* genes, we investigated sequence variations of all three *BrAOP2* genes in Yellow sarson type “R-O-18” which contains a high glucoraphanin content (3.006 ± 0.40 μmol g^−1^ DW) compared with those in Chines cabbage “Chiifu-401/42” with undetectable glucoraphanin. As a result, in the coding region of *BrAOP2.1*, two synonymous substitutions, two non-synonymous substitutions, a 3-bp deletion, and a 3-bp insertion were detected (Figure 1; Figure S3A). Although the C/A SNP at position +587 and G/C SNP at position +1,261 resulted in an amino acid change of T/N and E/Q, respectively, the change in T/N affected neither the amino acid R-chain charge nor the amino acid polarity and the change in E/Q did not belong to highly conserved amino acid residues, which are crucial for enzymatic activity in BrAOP2 (Zhang et al., 2015). Moreover, the 3-bp insertion (+564) and the 3-bp deletion (+604) resulted in a deletion and an insertion of amino acid, respectively, which is unlikely to have resulted in the functional change.

Interestingly, the B*rAOP2.2* gene sequence exhibited complete identity between the “R-O-18” and “Chiifu-401/42” with the exception of C/T SNP in exon 2 at the gene position +499 and G/A SNP at position +1128 (Figure 1; Figure S3B). The G/A SNP at position +1,128 resulted in a synonymous mutant, while the C/T SNP at position +499 led to production of a premature termination codon mutation.

Sequencing of the *BrAOP2.3* gene in “R-O-18” revealed four synonymous substitutions, four non-synonymous substitutions, and a 209-bp insertion in exon 2 at position +788 in the coding region (Figure 1; Figure S3C). Of these variations, the 209-bp insertion drew our attention because it resulted in a frame shift and generated a premature translation termination product.

Taken together, the sequence variation of *BrAOP2.2* and *BrAOP2.3* in “R-O-18” resulted in the premature translation termination product which most likely led to loss-function of the *BrAOP2.2* and *BrAOP2.3* genes, while the nucleotide variation of *BrAOP2.1* is unlikely to have altered the protein function in “R-O-18.”

### The *BrAOP2.2* and *BrAOP2.3* gene in Yellow sarson was non-functional

**Figure 2:**
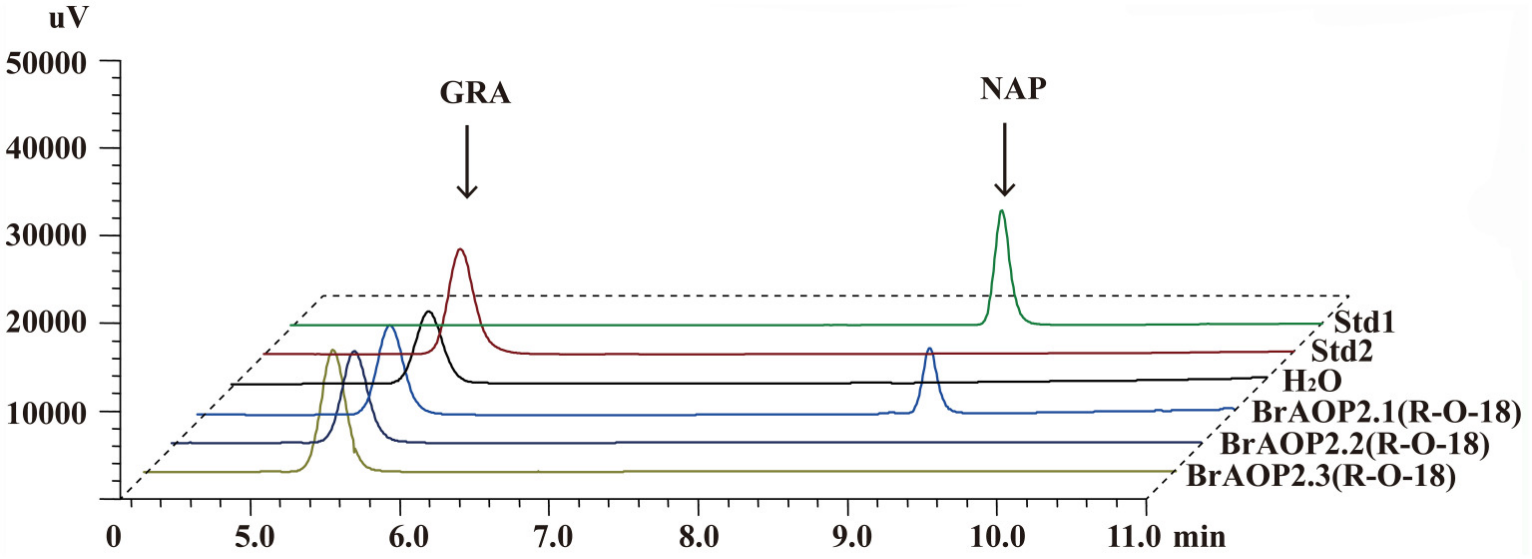
HPLC analyses of enzymatic activity of three BrAOP2s of R-O-18 *in vitro*. Std1 and Std 2 indicate desulfatedgluconapin (NAP) standard and desulfated glucoraphanin (GRA) standard, respectively. Conversion of GRA to NAP was catalyzed by BrAOP2.1, but not by BrAOP2.2 or BrAOP2.3 protein. H_2_O shows as the negative control.

Our previous study demonstrated that all three *BrAOP2* proteins in “Chiifu-401/42” could catalyze the conversion of glucoraphanin to gluconapin *in vivo* and *in vitro* (Zhang et al., 2015). To further determine whether the *BrAOP2.2* and *BrAOP2.3* in “R-O-18” are non-functional while *BrAOP2.1* is functional as predicted above, we analyzed the *in vitro* enzymatic activity of three BrAOP2 proteins in “R-O-18”. The full-length cDNA of the three *BrAOP2* genes were cloned from “R-O-18” and heterologously expressed in *Escherichia coli*. All the purified proteins (Figure S4) were incubated with the substrate glucoraphanin and we then tested their catalyze activity using enzymatic *in vitro* assays as described in Zhang et al. (2015). As shown in Figure 2, glucoraphanin can be converted to gluconapin by BrAOP2.1 protein. However, BrAOP2.2 and BrAOP2.3 protein were unable to catalyze glucoraphanin to gluconapin. The results indicate that BrAOP2.2 and BrAOP2.3 protein abolished catalysis activity, while BrAOP2.1 protein retained catalysis activity. This result was consistent with our prediction from the gene sequence analysis.

### Replacement of non-functional braop2.2 and braop2.3 increases the glucoraphanin content in *B. rapa*

To enrich accumulation of glucoraphanin in *B. rapa*, the non-functional *BrAOP2* genes were introgressed using marker-assisted backcrossing (MAB) strategy to a desirable *B. rapa* variety without glucoraphanin to replace the functional alleles. For this purpose, “R-O-18” containing high glucoraphanin content was used as a donor for the non-functional *braop2.2* and *braop2.3* allele, and “L58” containing undetectable or trace amounts of glucoraphanin harboring functional B*rAOP2.2* and *BrAOP2.3* which have the same coding sequences with “Chiifu-401/42” was used as a recurrent parent. The specific KASP marker for the *BrAOP2.2* gene (BrAOP2.2_KASP) and InDel marker for the *BrAOP2.3* gene (BrAOP2.3_InDel) were used to screen the heterozygous alleles (*BrAOP2.2/braop2.2, BrAOP2.3/braop2.3*) from the backcross populations (Figure S5). A total of 424 BC_1_ plants derived from “L58” × “R-O-18” were used for the first round of foreground selection (Figure S1) and 192 plants showed the homozygous genotype (*BrAOP2.2/BrAOP2.2*) for the BrAOP2.2_KASP marker, whereas 232 plants showed the heterozygous genotype. Then the 232 heterozygous plants at the*BrAOP2.2*locus were used for the selection of BrAOP2.3_InDel maker (Table S2). As a result, 112 plants showed the heterozygous genotype of these two alleles and then were used for subsequent background selection (Table S4).

**Figure 3:**
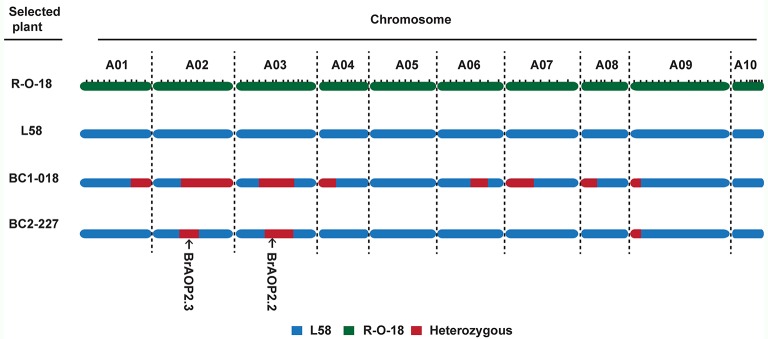
Graphical genotype of the selected BC_1_ and BC_2_plants. The green region represents segments from the recurrent parent “L58,” the blue region represents segments from donor parent “R-O-18,” and the dark red region represents heterozygous segments.

For background selection, the polymorphic InDel markers between parental lines “L58” and “R-O-18” were derived from the previous linkage map based on “L58” × “R-O-18” RIL population, which comprised a total of 372 InDel markers with a total length of 968.9 cM (Sun et al., 2016). Out of 372 InDel markers, 100 InDel markers were selected for background selection based on a physical map and linkage map, and then the recovery rates of the recurrent parent were calculated (Figure S2). The plant (BC_1_-018) with an 83.5% recovery rate were selected and crossed with the recurrent parent “L58” to produce BC_2_ plants (Figure 3). In total, 242 BC_2_ plants were obtained and used for the second round of foreground selection. Among these plants, 112 plants showed the BrAOP2.2/BrAOP2.2 genotype, whereas 130 showed the heterozygous genotype (BrAOP2.2/braop2.2). Then the 130 plants with the heterozygous genotype were retained for the selection of BrAOP2.3_InDel maker. A total of 64 plants harboring these two heterozygous alleles were used for background selection with 33 InDel markers, which were heterozygous in BC_1_-018. The plant BC_2_-227 with highest recovery rates (94%) was selected and selfed to produce BC_2_S_1_ (Figure S1; Table S4).

**Figure 4:**
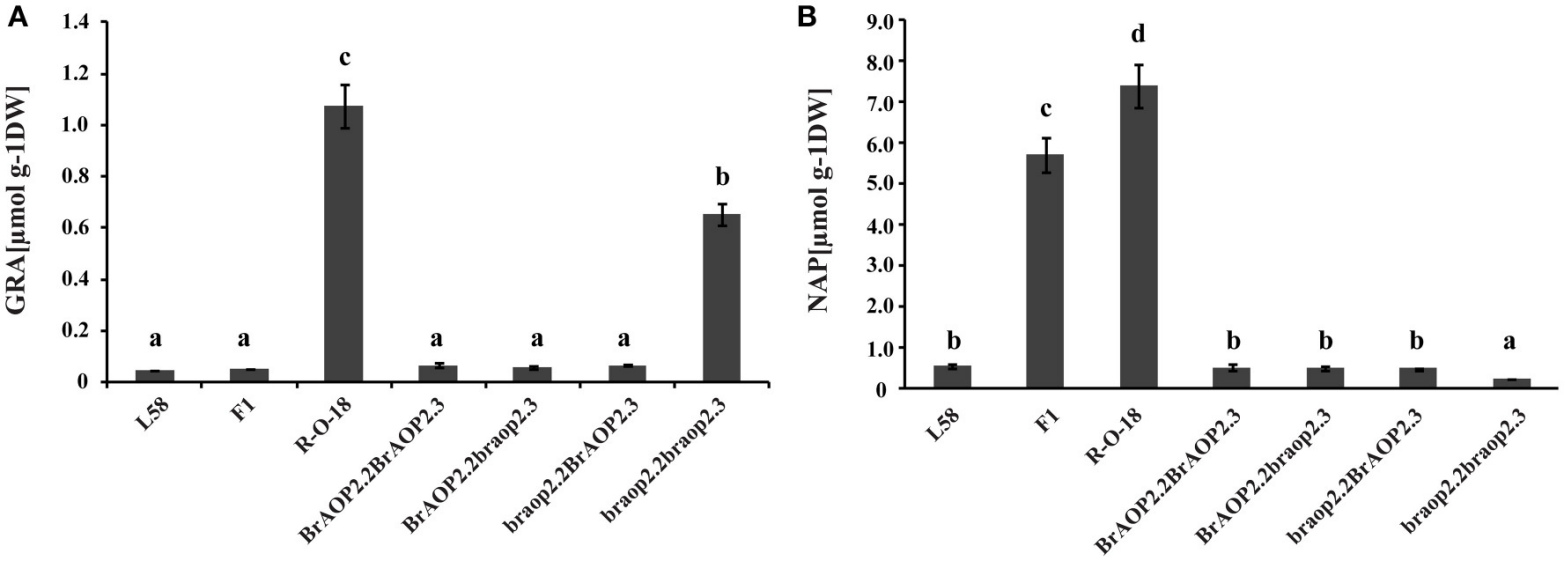
Glucoraphanin concentrations **(A)** and gluconapin concentration **(B)** in leaves of parental lines, F1 line and advanced backcross progenies. Data are shown as mean ± *SD* obtained from at least two biological replicates. Different letters indicate significant difference between genotypes (Tukey, *P* < 0.05).

The homozygous alleles of *braop2.2* and *braop2.3* were screened from BC_2_S_1_, and four types of homozygous combination alleles were obtained. These BC_2_S_1_plants were again selfed and generated BC_2_S_2_ plants (Figure S1). Of these, BC_2_S_2_ plants with homozygous combination were selected and their leaves were harvested to perform the glucosinolate analysis using HPLC. As shown in Figure 4A, individual lines with introgression of both *braop2.2* and *braop2.3* significantly increased the beneficial glucoraphanin concentration by 18 times compared with the recurrent parent “L58”. Further, significant reduction in the gluconapin was observed in the lines with *braop2.2* and *braop2.3* than recurrent parent “L58” (Figure 4B). However, the backcrossing progenies with introgression of single *braop2.2* or *braop2.3* did not change the levels of glucoraphanin or gluconapin. These results demonstrate that replacement of non-functional *braop2.2* and *braop2.3* can increase glucoraphanin accumulation content in *B. rapa*.

## Discussion

Glucosinolate biosynthesis is a rather complex process involved in more than 50 genes, forming various glucosinolates products from different precursor amino acid in Arabidopsis (Sønderby et al., 2010). Although both *Brassica* species and Arabidopsis belong to the Brassicaceae family, *Brassica* species experienced the event of whole genome duplication (WGD) from 10 million years ago, resulting in the expansion of gene number and gene divergence (Wang X. et al., 2011; Cheng et al., 2012). This caused the glucosinolate biosynthesis of *Brassica* species to be more complex than that of Arabidopsis.

Since all *Brassica* species undergoes gene duplication compared with Arabidopsis, it is difficult to screen the non-functional mutants with desired traits in *Brassica* species (Liu et al., 2012); that is, it is not easy to obtain loss-function of all functional redundancy genes. The RNAi or CRISPR/Cas9 systems have been demonstrated to be effective methods to knock down or knock out several homologous genes with sequence similarity to create the mutant with desired trait. To date, the RNAi has been successfully used to silence all the *AOP2* (*GSL-ALK*) genes, which resulted in the increase of beneficial glucosinolate glucoraphanin in *B. napus* and *B. juncea* (Liu et al., 2012; Augustine and Bisht, 2015). However, it remains challenging to increase the beneficial glucoraphanin content in *B. rapa* through knocking down or knocking out three *BrAOP2* genes using the RNAi or CRISPR/Cas9 strategies. This is because *B. rapa* is recalcitrant to Agrobacterium-mediated transformation (Vanjildorj et al., 2009).

The MAB method uses molecular markers to accelerate the selection of individuals containing the target locus from the donor line and high recurrence of the recipient background (Hospital and Charcosset, 1997). Compared with conventional backcrossing, MAB can save considerable time and labor for breeding new cultivars with desired traits. Therefore, pyramiding non-functional *BrAOP2* alleles using MAB seems an appropriate alternative method to replace all the functional *BrAOP2* genes in *B. rapa*. However, so far the non-functional *AOP2* alleles have only been found in some *B. oleracea*. Li and Quiros (2003) identified a 2 bp deletion in exon 2 of *BoGSL-ALK* (*BoAOP2*) in broccoli, which was demonstrated to abolish its function. Liu et al. (2014) identified two *BoAOP2* genes that were non-functional owing of the presence of a premature stop codon, which was suggested to contribute to the accumulation of glucoraphanin.

Although *B. oleracea* (*n* = 9, CC) is a close relative of *B. rapa* (*n* = 10, AA), the non-functional mutation of *BoAOP2* genes is difficult to use to replace the functional alleles of *B. rapa* by the interspecific hybridization between *B. rapa* and *B. oleracea*. In this case, the purpose of our current work was to identify mutants of non-functional *AOP2* genes from *B. rapa* accessions and then use the MAB method to pyramid all the non-functional *AOP2* genes into a target material and replace the functional alleles, thus inhibiting the conversion of glucoraphanin to gluconapin and increasing the glucoraphanin accumulation. However, we only identified natural mutant of non-functional *BrAOP2.2* and *BrAOP2.3* allele with premature termination in the Yellow sarson type containing high glucoraphanin, but not the non-functional *BrAOP2.1* allele mutant.

In order to introduce the non-functional *BrAOP2.2* and *BrAOP2.3* gene to the recurrent parent “L58” with undetectable glucoraphanin and functional *BrAOP2.2* and *BrAOP2.3* gene to increase the glucoraphanin concentration using MAB. 100 polymorphism inDel markers between “L58” and “R-O-18” were used for background selection, and the average marker density was ~9.68 cM. Previous report has shown that the density of markers for background selection were at least one every 10 cM (Herzog and Frisch, 2011). Therefore, 100 inDel markers were sufficient for MAB in our population.

The backcross progenies with introgression of both non-functional *BrAOP2.2*(*braop2.2*) and *BrAOP2.3*(*braop2.3*) alleles significantly increased the glucoraphanin accumulation compared with the recurrent parent, while replacement or introgression of a single *braop2.2* or *braop2.3* locus did not change the glucoraphanin content. These results demonstrated that loss-of-function of only one copy of *BrAOP2* did not affect their function of conversion of glucoraphanin to gluconapin because of the gene function redundancy. Accordingly, if all three provided *BrAOP2* genes were replaced by non-functional alleles, the plants would probably increase glucoraphanin accumulation as a result of the lower conversion of glucoraphanin to gluconapin relative to plants containing two non-functional *BrAOP2*. However, so far we have not identified the natural mutant of the *BrAOP2.1* gene in our collection of *B. rapa* accessions. Therefore, the non-functional mutant of *BrAOP2.1* should be screened, which could be achieved using the TILLING approach in a future study. In addition, we also noticed that introgression of two non-functional *BrAOP2.2* and *BrAOP2.3* leads to the increase of the glucoraphanin content without causing any obvious morphological appearance changes. These findings have profound implications for improvement of glucoraphanin-enriched *B. rapa* vegetable and oilseed crops, and the backcross progenies with high glucoraphanin content achieved in this study could be used to produce glucoraphanin-enriched *B. rapa* vegetables.

## Author contributions

ZL, JL, SZ, JZ, and FC performed the experiments and analyzed the data. ZL, JL, and XW wrote the manuscript. JW, WY, and XW conceived the project, designed the research and revised the paper.

### Conflict of interest statement

The authors declare that the research was conducted in the absence of any commercial or financial relationships that could be construed as a potential conflict of interest.
