# Supplementary material for: Enriching Glucoraphanin in Brassica rapa Through Replacement of BrAOP2.2/BrAOP2.3 with Non-functional Genes
Source: Front Plant Sci. 2017 Aug 2;8:1329. doi: 10.3389/fpls.2017.01329 (PMC5539120; doi:10.3389/fpls.2017.01329)
Supplement: Supplementary file 1 [file Table1.PDF]

Table S1 Composition and content (μmol g^{-1} DW) of glucosinolates in 70 *B. rapa* accessions.

Accession no.	Variety	Genotype	PRO	NAP	GBN	GRA	GBC	4ME	NEO	4OH
**DF14C001**	Z16	Chinese cabbage	1.12±0.06	0.16±0.01	0.7±0.05	nd	0.53±0.01	0.52±0.03	0.06±0.01	0.03±0.00
**DF14C002**	Ju Long	Chinese cabbage	1.00±0.21	0.76±0.08	0.99±0.07	nd	0.88±0.10	0.23±0.07	0.50±0.13	0.04±0.00
**DF14C003**	Xian Feng Xia Yang	Chinese cabbage	4.12±0.12	0.98±0.05	0.10±0.02	nd	0.65±0.20	0.45±0.04	0.08±0.02	0.11±0.02
**DF14C004**	BrIVFhn P1	Chinese cabbage	1.24±0.03	0.01±0.00	0	nd	0.69±0.06	0.80±0.04	0.11±0.04	0.11±0.01
**DF14C005**	BrIVFhn P2	Chinese cabbage	1.46±0.27	0.49±0.11	1.40±0.28	nd	2.02±0.24	0.36±0.02	0.36±0.12	0.03±0.01
**DF14C006**	V02A0704	Chinese cabbage	1.04±0.11	4.30±0.21	5.23±0.09	nd	1.75±0.12	0.27±0.08	0.17±0.03	0.04±0.01
**DF14C007**	Jian Chun	Chinese cabbage	1.32±0.06	0.06±0.01	0.06±0.01	nd	0.94±0.20	0.82±0.10	0.21±0.06	0.17±0.05
**DF14C008**	Shan Dong 4	Chinese cabbage	1.12±0.20	0.44±0.08	1.41±0.28	nd	1.80±0.09	0.51±0.03	0.10±0.02	0.15±0.01
**DF14C009**	Lu Bai 2	Chinese cabbage	0.89±0.16	0.32±0.14	0.49±0.06	nd	0.62±0.06	0.58±0.12	0.57±0.10	0.07±0.01
**DF14C010**	Ji Nan Da Gen	Chinese cabbage	0.96±0.09	6.12±0.28	3.96±0.17	nd	1.85±0.45	0.30±0.08	0.70±0.26	0.05±0.02
**DF14C011**	Da Tou Qing	Chinese cabbage	1.14±0.25	0.40±0.10	0.12±0.00	nd	0.84±0.11	0.45±0.04	0.15±0.01	0.03±0.00
**DF14C012**	Bei Jing 3	Chinese cabbage	1.08±0.34	0.11±0.02	0.51±0.06	nd	1.41±0.13	0.31±0.00	0.14±0.05	0.06±0.01
**DF14C013**	Zao Shu 50	Chinese cabbage	1.19±0.09	0.07±0.01	0.18±0.06	nd	0.39±0.12	0.25±0.04	0.06±0.00	0.18±0.04
**DF14C014**	L488-3	Chinese cabbage	1.02±0.17	2.20±0.11	3.25±0.20	nd	2.20±0.32	0.25±0.05	0.28±0.07	0.12±0.04
**DF14C015**	33--3	Chinese cabbage	0.71±0.04	0.28±0.07	0.62±0.08	nd	1.43±0.10	0.21±0.06	0.25±0.02	0.20±0.05
**DF14C016**	Hua Bai 2	Chinese cabbage	0.70±0.07	0.04±0.01	1.28±0.21	nd	0.57±0.12	0.41±0.03	1.24±0.23	0.07±0.02
**DF14C017**	V02A1499	Chinese cabbage	1.16±0.26	0.17±0.06	0.42±0.04	nd	0.43±0.02	0.37±0.03	0.19±0.04	0.04±0.00
**DF14C018**	V02A1396	Chinese cabbage	0.71±0.09	0.01±0.00	0.13±0.02	nd	1.77±0.12	0.31±0.06	1.07±0.12	0.03±0.00
**DF14C019**	CGN07146	Chinese cabbage	0.85±0.12	0.29±0.06	0.82±0.06	nd	0.27±0.08	0.22±0.03	0.22±0.02	0.09±0.02
**DF14C020**	Xin Lv 85	Chinese cabbage	0.66	0.21	0.11	nd	1.94	0.41	0.42	0.08
**DF14C021**	328	Chinese cabbage	0.67±0.04	0.90±0.03	2.54±0.32	nd	1.16±0.09	0.08±0.01	0.10±0.03	0.30±0.08

Continued

**DF14C022**	Xia Kang 40	Chinese cabbage	0.92±0.20	0.03±0.01	0.31±0.05	nd	0.14±0.03	0.40±0.04	0.12±0.03	0.03±0.00
**DF14C023**	Zhu Long Cai	Chinese cabbage	1.25±0.06	0.07±0.02	0.22±0.04	nd	0.66±0.03	0.15±0.02	0.12±0.00	0.28±0.04
**DF14C024**	Qin Bai 5-4-2	Chinese cabbage	0.57±0.05	0.16±0.03	0.88±0.05	nd	1.23±0.06	0.35±0.02	0.19±0.00	0.01±0.00
**DF14C025**	Shi Te	Chinese cabbage	0.97±0.03	0.73±0.05	1.56±0.07	nd	1.22±0.22	0.31±0.03	0.63±0.09	0.08±0.01
**DF14C026**	V02A0555	Chinese cabbage	0.66	0.17	0.09	nd	0.28	0.15	0.12	0.04
**DF14C027**	Yun Hong Zhong Jiang	Chinese cabbage	0.91±0.08	0.06±0.01	0.02±0.00	nd	0.20±0.02	0.07±0.01	0.38±0.03	0.46±0.10
**DF14C028**	Da Qing Ma Ye	Chinese cabbage	3.14±0.24	0.11±0.01	0.64±0.10	nd	1.34±0.10	0.41±0.06	0.14±0.05	0.09±0.01
**DF14C029**	Si Ji Chun	Chinese cabbage	0.97±0.09	0.33±0.04	0.21±0.04	nd	0.38±0.04	0.57±0.11	0.21±0.01	0.07±0.01
**DF14C030**	CGN06688	Chinese cabbage	1.88±0.23	0.10±0.02	0.59±0.09	nd	1.30±0.09	0.46±0.03	0.61±0.10	0.31±0.04
**DF14C031**	SynBr01	Chinese cabbage	3.01±0.03	1.35±0.01	4.49±0.03	nd	0.39±0.01	0.28±0.01	0.23±0.02	0.26±0.00
**DF14C032**	SynBr03	Chinese cabbage	1.04±0.01	3.32±0.14	0.40±0.08	nd	1.02±0.10	0.79±0.02	0.09±0.01	0.24±0.02
**DF14C033**	V02D0130	Caixin	2.78±0.40	11.13±2.21	0.79±0.10	nd	0.20±0.06	0.15±0.04	0.30±0.07	0.05±0.00
**DF14C034**	70 day You Qing	Caixin	0.04±0.00	0.78±0.05	0.32±0.06	nd	0.16±0.02	0.07±0.00	0.01±0.00	0.02±0.00
**DF14C035**	L58DH	Caixin	0.23±0.03	5.00±0.22	1.10±0.07	nd	0.40±0.02	0.15±0.05	0.24±0.04	0.08±0.00
**DF14C036**	L41	Komatsuna	0.65±0.10	10.34±1.25	8.26±0.81	nd	0.58±0.10	0.11±0.00	0.43±0.03	0.10±0.02
**DF14C037**	CGN17281	Komatsuna	0.55±0.12	10.07±1.12	0.80±0.06	nd	0.21±0.05	0.12±0.02	0.18±0.03	0.04±0.01
**DF14C038**	Shang Hai Qing	Pakchoi	1.00±0.08	3.46±0.06	2.00±0.30	nd	0.17±0.02	0.20±0.00	0.10±0.02	0.02±0.00
**DF14C039**	V02B0645	Pakchoi	0.98±0.05	5.03±0.35	1.19±0.20	nd	0.26±0.05	0.06±0.00	0.2±0.03	0.12±0.04
**DF14C040**	Hei You Bai Cai	Pakchoi	0.55±0.20	1.71±0.10	0.33±0.04	nd	0.42±0.10	0.08±0.02	0.12±0.01	0.08±0.02
**DF14C041**	Gao Jiao Shi Geng Bai	Pakchoi	1.08±0.02	0.17±0.00	0.27±0.11	nd	0.3±0.00	0.23±0.09	0.43±0.06	0.06±0.01
**DF14C042**	Zhong He Qing Cai	Pakchoi	0.46±0.10	0.12±0.01	0.07±0.01	nd	0.10±0.02	0.04±0.00	0.25±0.06	0.02±0.00
**DF14C043**	Su Zhou Qing	Pakchoi	0.96±0.02	2.77±0.24	1.60±0.13	nd	0.25±0.03	0.20±0.04	0.05±0.01	0.03±0.00

Continued

**DF14C044**	Lv Xin Qing Cai	Pakchoi	1.01±0.04	1.44±0.20	0.10±0.01	nd	0.51±0.05	0.05±0.01	0.19±0.01	0.25±0.00
**DF14C045**	Si ji Xiang Cui	Pakchoi	0.69±0.03	0.68±0.00	0.63±0.05	nd	0.45±0.08	0.07±0.02	0.2±0.07	0.15±0.03
**DF14C046**	Jin Sha Qing Jiang	Pakchoi	0.94±0.12	1.79±0.07	0.19±0.01	nd	0.26±0.04	0.28±0.08	0.23±0.09	0.02±0.00
**DF14C047**	Si Yue Qi	Pakchoi	0.78±0.11	3.63±0.16	4.24±0.49	nd	0.29±0.09	0.16±0.05	0.10±0.01	0.02±0.00
**DF14C048**	Wu Yue Man	Pakchoi	0.91±0.10	6.72±0.46	0.26±0.04	nd	0.16±0.02	0.20±0.05	0.26±0.06	0.01±0.00
**DF14C049**	V02B0591	Pakchoi	0.82±0.07	0.74±0.07	0.24±0.02	nd	0.34±0.07	0.07±0.01	0.23±0.07	0.17±0.03
**DF14C050**	Hua Guan Qing Geng	Pakchoi	1.08±0.02	7.32±0.44	0.77±0.06	nd	0.14±0.00	0.19±0.01	0.09±0.03	0.05±0.00
**DF14C051**	V02B0002	Pakchoi	0.64±0.10	1.27±0.29	0.31±0.01	nd	0.32±0.10	0.07±0.01	0.34±0.04	0.18±0.07
**DF14C052**	CGN06790	Mizuna	0.34±0.05	24.3±2.03	1.26±0.12	nd	0.30±0.04	0.11±0.02	0.16±0.04	0.05±0.01
**DF14C053**	CGN17279	Mizuna	0.40±0.06	12.7±0.90	1.11±0.18	nd	0.47±0.07	0.23±0.01	0.41±0.04	0.10±0.01
**DF14C054**	Shao Zi Tou	Taicai	0.90±0.09	1.64±0.05	2.66±0.49	nd	0.51±0.05	0.12±0.02	0.28±0.02	0.07±0.01
**DF14C055**	V02C0008	Taicai	1.89±0.40	2.42±0.39	2.31±0.03	nd	0.84±0.09	0.11±0.02	0.74±0.07	0.28±0.06
**DF14C056**	V02C0012	Taicai	1.08±0.11	4.38±0.42	4.21±0.07	nd	0.27±0.01	0.11±0.00	0.31±0.02	0.19±0.04
**DF14C057**	Zao Zhuang Xiao Ma Ye	Taicai	2.63±0.08	3.99±0.42	5.39±0.06	nd	0.28±0.03	0.06±0.00	0.93±0.04	0.20±0.02
**DF14C058**	V01C0036	Turnip	0.80±0.00	28.5±3.29	5.81±0.11	nd	0.49±0.10	0.04±0.00	0.35±0.04	0.13±0.03
**DF14C059**	CGN15220	Turnip	0.20±0.02	34.43±1.61	13.07±2.12	nd	0.51±0.05	0.32±0.07	0.24±0.01	0.11±0.03
**DF14C060**	CGN15201	Turnip	0.37±0.03	25.94±2.05	5.72±0.12	nd	0.38±0.08	0.08±0.00	0.36±0.02	0.13±0.02
**DF14C061**	CGN06721	Turnip	0.41±0.09	9.45±0.91	5.35±0.20	nd	0.47±0.04	0.14±0.02	0.23±0.03	0.17±0.07
**DF14C062**	Lv Ling	Wutacai	0.76±0.05	4.31±0.17	3.80±0.26	nd	0.71±0.09	0.11±0.01	1.77±0.48	0.06±0.01
**DF14C063**	Zhong Ba Ye	Wutacai	0.66±0.10	0.39±0.05	0.24±0.05	nd	0.71±0.12	0.09±0.01	0.26±0.08	0.09±0.01
**DF14C064**	SynBr01 (R-O-18)	Yellow sarson	0.65±0.11	23.24±1.26	0.16±0.02	3.01±0.39	0.07±0.02	0.06±0.02	0.13±0.03	0.01±0.00

Continued

**DF14C065**	L143	Yellow sarson	0.49 ±0.01	22.57 ±3.77	0.40 ±0.05	0.99 ±0.28	0.08 ±0.00	0.08 ±0.01	0.20 ±0.01	0.02 ±0.01
**DF14C066**	SynBr02	Yellow sarson	0.65 ±0.03	23.08 ±3.20	0.12 ±0.01	4.63 ±1.87	0.12 ±0.02	0.07 ±0.01	0.08 ±0.02	0.04 ±0.01
**DF14C067**	V02D0190	Zicaitai	0.27 ±0.03	0.35 ±0.06	2.43 ±0.11	nd	0.48 ±0.30	0.13 ±0.01	0.23 ±0.09	0.16 ±0.03
**DF14C068**	Zicaitai	Zicaitai	0.08 ±0.01	3.57 ±0.28	1.64 ±0.24	nd	0.06 ±0.02	0.02 ±0.00	0.02 ±0.00	0.07 ±0.01
**DF14C069**	Shi Yue Hong	Zicaitai	0.24 ±0.07	3.88 ±0.32	3.63 ±0.30	nd	0.19 ±0.02	0.25 ±0.05	1.18 ±0.17	0.19 ±0.03
**DF14C070**	SynBr05	Zicaitai	1.25 ±0.16	0.18 ±0.01	0.46 ±0.10	nd	0.10 ±0.00	0.10 ±0.00	0.09 ±0.01	0.05 ±0.00

Table S2 Primer sequences of the BrAOP2.2_KASP and BrAOP2.3_InDel markers.

	Forward primer		Reverse primer
	FAM-labeled primer	Hex-labeled primer	
BrAOP2.2_KASP	GAAGGTGACCAAGTTCATGCTGGTGCTGAATACTTCATCAGTCG	GAAGGTCGGAGTCAACGGATTGTGGTGCTGAATACTTCATCAGTCA	AGAACCTCAAGTCAATGAATTACCGTCTA
BrAOP2.3_InDel	GTGCTGGTGATGGTGCTAATGATG		CAACACCAGCACTACTGAGAGTAC

Underline represents the common primer of FAM and Hex, respectively.

Table S3 The details of 100 InDel markers distributed across 10 chromosomes.

Marker	Chromosome	Physical position	Forward primer	Reverse primer
BrID101093	A01	2953247_2953356	TAAAAACTTCCCAACAGCC	TATGGAATTAATCGCGCAC
BrID10303	A01	6173728_6173808	GAATACTTACCCCGTTGTTG	CTACACCGGTTTTTAAAGTG
BrID11389	A01	9518833_9518921	ACGGACGTCTAAACCGTATT	GGCTACTCCATACAAATTGC
BrID10769	A01	11126593_11126658	TCTGACTTTCTAGCCACGAT	CAGATGGTAAACATGCTGC
BrID11071	A01	13471226_13471326	CTTCCCATGATTTTCTTCCT	TGAGAGTGAGTTCCTCTTCTT
BrID11075	A01	15445787_15445870	GATCAAACAGAATAGCCTCG	TCTGCTTCGAGTTCAAAGAT
BrID11081	A01	17477896_17477996	CTCCTGGTGAATCCTCTTTT	TATCTGCATGAACGAAGTTG
BrID11345	A01	20460348_20460438	GCTTATGGTGAAGATCATGG	TAGGCTACGGGTAGAGCATA
BrID11385	A01	23532386_23532486	ATAGGAAGCAGACCATGAAA	TCCGAATCCTAACCATACAG
BrID11087	A01	26741675_26741774	ATGGTCATCTCCTACAAACG	CTCACTTTCTCATGCACCTT
BrID11131	A02	618261_618360	CTTCAGAGCATTTCCTCAAT	ACCTTAAAACAAATCCAGGC
BrID11431	A02	2921994_2922086	CCATACACCGATTTCTTGTT	GTCTCTTTTTCTTTCCGGTT
BrID11113	A02	7096627_7096727	GTCTCGATTTCTTCAGCAAC	CAATCTTCAGAACCGTGACT
BrID10199	A02	10866494_10866621	CTGGTTGGGTTTTGTGTTAT	GCCATGCAAAATACTACCAC
BrID10555	A02	13057839_13057918	TGACTTTGGTTCTCAAATCC	ACTTCTCCACCAGTCCATAA
BrID11895	A02	16722592_16722681	TCGGAGATTCTGGAACTTTA	GCTATTTTGATGCTAAACGG
BrID11909	A02	19790230_19790286	GTCAGGACTACAAATCGCAT	CTAATGTGTCATTTGCATGG
BrID101131	A02	22083847_22083945	CAAAAAGTCCACGAAACATC	GGATGCCATTCCTCCTATAA
BrID11097	A02	25331488_25331552	TAACTGTTGGCTTCTCACCT	TAGTATCATTTGTGGCATGG
BrID11091	A02	26089645_26089745	CCTCGTCAACAACAACTACA	TGGTGTTGAAACTGATTCTG
BrID10151	A03	704674_704804	CCAGTGTTTCTCGTTTGAGT	GACACTATGCTCTGGCTGAT
BrID11663	A03	2174662_2174750	AATGGACTAGTTGCTGCTTC	AGCCCCTGGAAAGTAATAGA

Continued

BrID10705	A03	5624844_5624917	ATTCGAGGACTTGAGTAGCA	GTTCCACGTCAGCAAATAC
BrID10027	A03	8861844_8861980	AATAGACCGGTAAAACTTGC	CCCATTAACTCACTCACACA
BrID90412	A03	11353513_11353691	AGGACGCTTGCACGTTTAGT	CGGTCGTTGGTTAGTTAGGTG
BrID90420	A03	13609131_13609288	TTTTTCTTCCTTTGGCTTCTTT	CATCTCTAAGGCTTGAACAAAATG
BrID10461	A03	16564624_16564701	TAAGATAATGGAACGATGGC	CTAATCGTTGTCATGAATCC
BrID10063	A03	18010531_18010657	GCGTCAGTTTATGGATCTTC	GTGACACTAAAATGAAGGAGG
BrID90027	A03	21569444_21569624	GTGATTATTGGCATTCGTCG	TCCCACTTTGCAAGATCTCC
BrID90373	A03	24100828_24100963	AGCCACAATTAAGCCACGTT	TTGTGCATCTATTGAGTAGGGC
BrID11145	A03	26806603_26806688	CAAGGTTTTGAGCCTTACAC	TCCCTTTCACCAATAACATC
BrID11141	A03	28148960_28149016	ATCAGGCAACCTAAGATCAA	TGTCAACTGTGGAGACAGAA
BrID11135	A03	29165637_29165716	TTCTCTTCTTCAAGCGATTC	GGAAGAGAACAAACATGAGG
BrID90059	A04	89767_89934	TTCATGCTTACCGACACTGG	TGGGACACCTTGTGTTCTTG
BrID101473	A04	2490718_2490815	TCCCAGTTGCTCAATATGAC	CTCTGAACCAAAAAGATGAC
BrID101495	A04	3368222_3368319	GTATTTTGGGAGGCTTCTTT	AGACTCCAGCATATTGTCTATC
BrID101451	A04	5485731_5485842	CGTTAACCGTTCCTTTTTAC	CGCCGATAATTTGTTTACTG
BrID10639	A04	8367355_8367432	CGAGTTATTGGTTAGAATCG	TTTATTTTCCGATAGAGGGG
BrID11157	A04	10057988_10058088	CTTAGGCTCCTCAAGTTTCA	CTCTTACATAAGCCACCGTC
BrID90295	A04	14154965_14155133	CACAAAGCTCACTTCTTAGGCA	TATAGCCTTGCGGTGGAGTT
BrID11035	A04	15985690_15985766	CTAATAACGTGGCAGCTCTT	GTCGATGTGTTTTTGTTGC
BrID11453	A04	17799313_17799363	GTTTAACAATGTATGTGGGG	AGAAAATTGACAAGAGCGTC
BrID11623	A05	57480_57573	AGCTAAAGAGAAAGGGAAGG	GACTTTACCAATTCCAGTCG
BrID10135	A05	2864519_2864653	CGCTAAAAATAACTGAACCG	CTAATGTTTGGGATTTACCC
BrID11641	A05	5517399_5517499	GAACAGAAACAGCATTACCC	TTATTGTTCCTCTGGTTCGT
BrID90337	A05	10853115_10853279	AGTCCATACGCCATTGCTTC	TCGGTTTTGATTTCAGATTGATT
BrID101239	A05	13341296_13341412	TCCACACAGAAGATAACTGGT	GAGATGACATTTTCGCTGAC

Continued

BrID90355	A05	15522085_15522256	GGGAGGATCCACAAGAAAATC	GGTCTTGATAGAGTTGCGCC
BrID11753	A05	18911877_18911972	GTTGGTTGGTTGTTTGAATC	TATGGAAACACGAACGAGTA
BrID11165	A05	20668083_20668178	AGTGATGGGAAGAGAAAACA	CATCTCTCGCTCTACCACTC
BrID11327	A05	22920362_22920417	GATAAACTTTGCATGGTTGC	GACTGATTTCGCATGATCTC
BrID101173	A05	24638046_24638127	CTCCTCCTTTCGTAATGAAT	CAACGAAGAAGATAGAGGC
BrID11183	A06	114736_114832	CGTTTAATGATCCTTTCGAG	GGCCTAAACAGATGAGATGA
BrID111045	A06	3285644_3285743	TCCAAGATTGGAGTTGAAGT	ACAAGTCTCCTCCTAGCTCC
BrID10649	A06	7374429_7374489	CTGTGTCTTGAGGATTAGAGG	AGAGCATCAGTTGTTTTTGG
BrID10381	A06	10324718_10324797	CATGTAAGTCTGCCCTTTTT	TGTTGAAATCTGGTAGGAAC
BrID11015	A06	14029392_14029475	CTATTGATTTGGTGTGGCTT	TAAAGTGAAACCCAAAGCTC
BrID11205	A06	17495779_17495844	AGATTTAACACCATAGCCCA	CGTCGTCCTTTACTTTTGAC
BrID10257	A06	19048749_19048824	CAGAAACATCACTGGGTCTT	GCTGTTTGACCTGATATCCT
BrID11487	A06	22609597_22609689	ATCATATCGCAGAGCTTCAT	GCGTATATATAATGCCACCC
BrID11225	A06	24841747_24841823	AAACGACCTTTGTCCTCAAT	CCCTAATTCAGATTTTCGTG
BrID101139	A07	648999_649117	ATTGAAGCAGTGGTGTGCT	GAAACAAAGCATTGTCACAC
BrID11023	A07	4778740_4778809	CATTCCACCCACAACTTTAG	TCAACAGATCAGTGGATGAA
BrID111245	A07	6438435_6438508	TATTCCATTCGTCTTCAACC	CTGTCTCAAATGTTGATCCTC
BrID90051	A07	10727465_10727638	TAGCATAGCAATAATGACAAA	TCAAATCTCCAAACAATCC
BrID11587	A07	14793428_14793526	TGCTGTTGATCAAGAAAGAC	CTTGCTCTCCTTCACCTTC
BrID101029	A07	16613449_16613553	GCCAATATCTCCAGTTTCAT	TGTTTGGTTTCATCTGACTG
BrID11607	A07	18359119_18359182	CAACATAACCCATCACCTCT	GGAATTAGGATTTAGGACTGC
BrID11615	A07	20240840_20240913	ATGACTGTTAGGAATCCCAT	GCTAATACATGAATCCCTCG
BrID90109	A07	22970737_22970901	AAGGATCTTTGGCGATTGTG	ACCCTACCCATCGTCTTCCT
BrID90189	A08	150343_150498	GGAGTACCTGAATCCAACATCA	CACAATCTTAATCCTCGGGAA
BrID10927	A08	2325294_2325396	CGATATCGTATAGGACCTTCA	CTTCATTCAATGCTGATGC

Continued

BrID10877	A08	5966085_5966156	GCTCATGACCTCAGACTTATG	GAGCGTGTTATCTCTCTTGC
BrID10427	A08	10654271_10654338	TTTGTTCTCTTCTTCCTTCG	CGACAAGATAAGGGAATCTG
BrID11239	A08	12027707_12027798	GATTTCATGATGCTTGGTCT	ACAGAAACAACATTTCCTGC
BrID11685	A08	14490617_14490711	TACTTGCGTCAGCCATTAG	TGAGTCTTGTGTCAATGTCG
BrID11717	A08	17118775_17118832	GCTTAATGTATGGAATGGTG	CAGTAACTGCTAACGCCACT
BrID111405	A09	748984_749068	GCAAAATGCACAACAAACAC	TTCTATACTGGAGCAATGCC
BrID90363	A09	2284069_2284228	TTCTGTTTCGAAAAATGCCA	TGTTCTTGCCAAGTCTCACG
BrID111395	A09	4008619_4008706	TTCCGTTACTCCAATCTGAC	TGAAGATCGGTCTCAAAGTT
BrID111055	A09	7565208_7565294	CGATGTTTGGTGTTACTCAA	GCTGCTGGAGTTATACCTTG
BrID11271	A09	9757016_9757105	AAGTGCGGTTCTGTTACTGT	GTGAAGACATCATCAATCCC
BrID11535	A09	12139964_12140063	GTTATGTATGGCAGGTGGTC	GCTCTCTGGATGGTTTATTG
BrID101055	A09	18221869_18221986	AGACTTCAGCGGTTTGAATA	GGGTCTAGGCGAAAAATTG
BrID11261	A09	21434183_21434253	GAGAAAAGCTCTTGCAGAAA	GAGGTCCAGAACCTTTTACC
BrID101175	A09	23804590_23804671	GGTCAATGAAGGAGCTTGT	GAAGAAGAGACAAATCTACGGA
BrID111407	A09	27271120_27271219	CTCCCTTCAAAAACAATGAG	TCGATGGAGAAGGACATTAC
BrID10187	A09	30097858_30097980	GCCATAAGTTAGGCAAAAAG	CACGTGATTTGGTAAATTCC
BrID11781	A09	33764991_33765059	AGAATAAATGACACCAGCGT	CATTTTGAGGGTCATTCTCT
BrID11285	A09	36872678_36872743	GGTTTTGTTGAAAGAGTTGC	CAGAGTTCCGGTTTTATCAG
BrID11289	A09	38251657_38251756	GAAAGGTGGCAAACTTAATG	ACTGTCACCAAACAAAGAGC
BrID11027	A10	1157030_1157100	CCACTTGCTTTGAGTATTCC	GTTTTGGTTGTGTCACCTCT
BrID11579	A10	3588508_3588598	GTTTTAACGAGGCTAAGGGT	GCTGAGAGTTCAGCACAAGT
BrID11549	A10	5459116_5459213	GTATATCTGAGAGGCAACCG	AACCTAACAAAGCAAAGACG
BrID11003	A10	8583268_8583366	CAATAGATTTATGCAGGGGA	TTACGCTTCCATTGTTCTAC
BrID11539	A10	11177793_11177885	TTCAAGAAGAGGAAGAACCA	TTTGGTCTTTCTCCAACCTA
BrID11551	A10	12772418_12772496	GCTATGACTGTTTTTGGCTT	TACCTAACGCTGAACTGATG

Continued

BrID10569	A10	13833054_13833127	CATCTTTCTTCTCACACATCAC	GGTAGAGGAGAGGTTCCTTG
BrID90139	A10	14982106_14982242	TGAGCATGAGTTTGTCACTGC	GCAACATTGTGAATTGGCTG
BrID11011	A10	16256274_16256338	CAGCTAAAGCTGAAGATTGC	TGTGAAAGGAAAGTCTCAAC

Table S4 Genotyping results of the foreground selection in the ‘L58’ × ‘R-O-18’ backcross population

Population	Generation	Alleles	Number of plants			Expected ratio	P value
			Total	Heterozygous alleles	Homozygous alleles		
‘L58’ ×‘R-O-18’	BC1	BrAOP2.2	424	232	192	1：1	0.0521
		BrAOP2.3	232	112	120	1：1	0.5994
	BC2	BrAOP2.2	242	130	112	1：1	0.2472
		BrAOP2.3	130	64	66	1：1	0.8608
