# Supplementary material for: Enriching Glucoraphanin in Brassica rapa Through Replacement of BrAOP2.2/BrAOP2.3 with Non-functional Genes
Source: Front Plant Sci. 2017 Aug 2;8:1329. doi: 10.3389/fpls.2017.01329 (PMC5539120; doi:10.3389/fpls.2017.01329)
Supplement: Supplementary file 2 [file Image1.PDF]

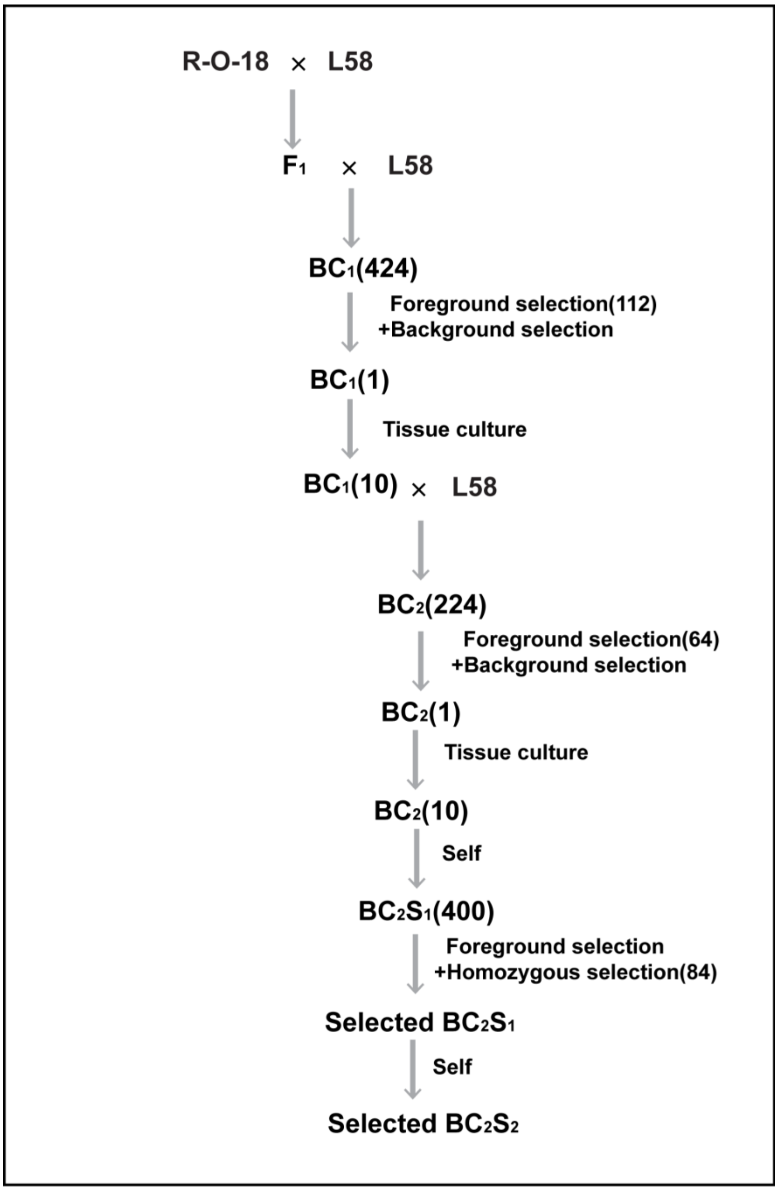

Figure S1 Schematic diagram for introgression or replacement of non-functional *BrAOP2* alleles in ‘L58’ from ‘R-O-18’. The number in parentheses indicates the plants selected in each generation.

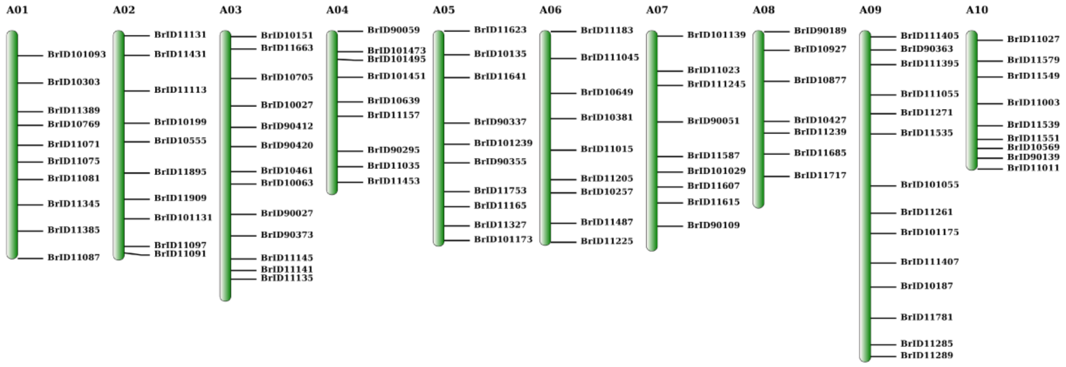

Figure S2 Distribution of 100 polymorphic InDel markers in 10 chromosomes of *Brassica rapa.* The represented distances are based on a physical map.

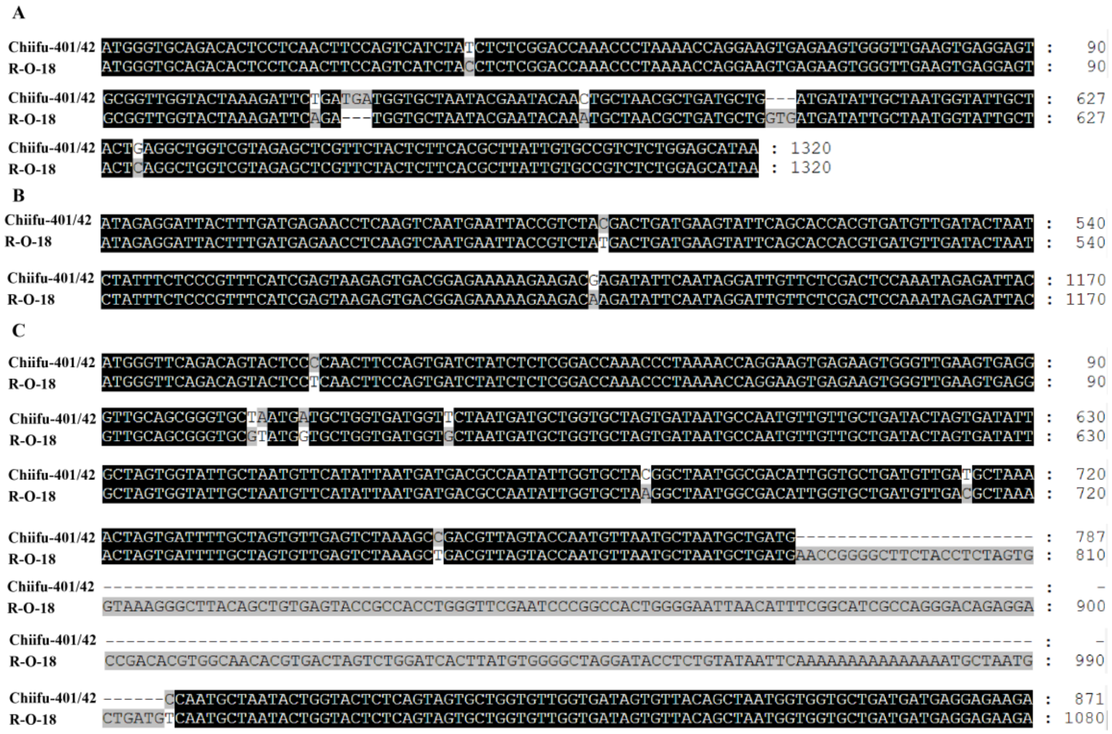

Figure S3 Nucleotide sequences alignment of coding DNA sequences (CDS) of three *BrAOP2* genes between ‘Chiifu-401/42’ and ‘R-O-18’. Only regions containing variations in *BrAOP2.1*(A), *BrAOP2.2*(B) and *BrAOP2.3*(C) are shown. Pairwise alignments were performed using MEGA v.5.0. Dark shading represents conserved residues.

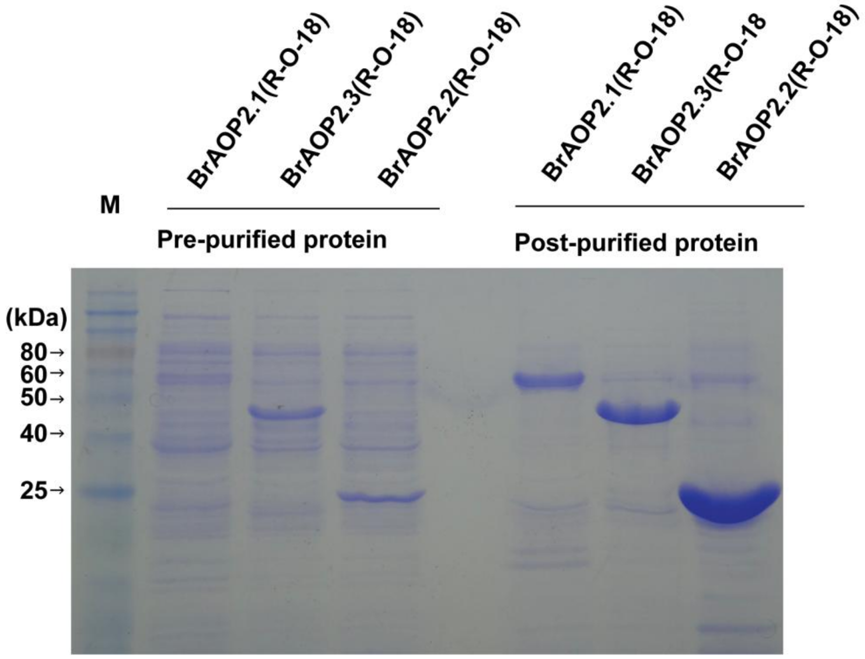

Figure S4 Purification of three BrAOP2 proteins from ‘R-O-18’. SDS-polyacrylamide gel electrophoresis (SDS-PAGE) and Coomassie Brilliant Blue staining of His-tagged BrAOP2 proteins before purification (Lanes 2–4) and after purification (Lanes 5–7) with an Ni-NTA His Bind Purification Kit. Lane 1, molecular mass standard.

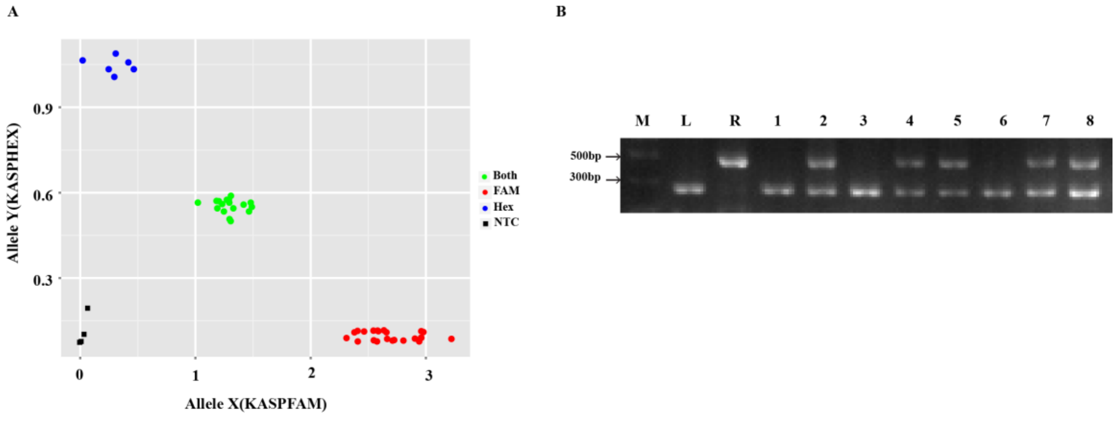

Figure S5 Genotyping of *BrAOP2.2* by KASP assay and *BrAOP2.3* by an InDel marker in BC1 population. (A) BrAOP2.2_KASP genotypes in 40 individuals randomly selected from BC1 population and six replication of 'R-O-18'. Allele X (KASPFAM, red) shows the C nucleotide, and allele Y (KASPHEX, blue) shows the T nucleotide. The black box represents the water control. (B) The PCR products were amplified by the BrAOP2.3_InDel marker in 8 individuals randomly selected from BC1 population. L and R represent 'L58' and 'R-O-18', respectively.
